# Timing of treatment with oral propranolol for infantile hemangioma

**DOI:** 10.1186/s12887-025-06033-5

**Published:** 2025-09-24

**Authors:** Kaizhi Zhang, Tong Qiu, Jiangyuan Zhou, Xue Gong, Zixin Zhang, Shanshan Xiang, Yi Ji

**Affiliations:** Division of Oncology, Department of Pediatric Surgery, West China Hospital of Sichuan University, #37# Guo-Xue-Xiang, Chengdu, 610041 China

**Keywords:** Infantile hemangioma, Propranolol, Treatment, Timing

## Abstract

**Background:**

Early oral propranolol for infantile hemangioma (IH) can improve the success rate. However, few studies have been conducted on the specific age threshold for initiating treatment with propranolol. This study aimed to determine the cutoff value of treatment success for IH with oral propranolol.

**Methods:**

This was a retrospective study involving 207 patients with IH and clinical data.The primary outcome measure was treatment success with oral propranolol at months 6 to 12.

**Results:**

Multivariate analysis showed that age at treatment initiation (*P* < 0.001), high-risk IH (*P* = 0.002), and segmental IH (*P* = 0.012) were independent risk factors for treatment unsuccess with oral propranolol at months 6 to 12. Receiver operating characteristic (ROC) curve analysis indicated that the cutoff value for age at treatment initiation was 69.5 days for all patients, 65.5 days for patients with segmental IH or high-risk IH, and 93.5 days for patients with non-high-risk and nonsegmental IH. Patients who started treatment before 69.5 days had a success rate of 73.8%, which was higher than the 28.4% success rate of patients who started treatment after 69.5 days. The median time to treatment success for patients who started treatment before 69.5 days was 11.5 months, which was shorter than the 15 months for patients who started treatment after 69.5 days.

**Conclusion:**

The initiation of treatment for IH with oral propranolol before 70 days of age can improve the success rate and shortens treatment duration, especially for segmental IH and high-risk IH.

**Clinical trial number:**

Not applicable.

## Introduction

Infantile hemangioma (IH) is the most common benign tumor in infants, with an incidence of about 4.5%, and it occurs significantly more often in females [1–3]. The majority of IHs emerge at birth or within one week after birth [4]. Currently, IHs can be categorized into superficial, mixed, and deep types and can also be divided into localized, indeterminate, and segmental subtypes on the basis of morphological subtype [5]. IH is marked by rapid initial growth, followed by a slower resolution phase. Approximately 80% of cases reach their maximum size at 3 months after birth. Most IHs resolve spontaneously and persist for several years. A small number of patients with IH may develop severe complications, including disfigurement and ulceration. IHs that grow in the airway or eyes can be life-threatening [6, 7].

In 2008, Léauté-Labrèze et al. [8]initially identified propranolol as an effective treatment for IH. Since then, propranolol has become a first-line treatment for IH [9]. Oral propranolol before 3 months of age in patients with IH has been reported to improve prognosis [10]. The clinical practice guideline for the management of IH suggests that the ideal age for IH treatment is 1 month [11]. However, few studies have verified the recommendation, and the timing of oral propranolol for IH remains worthy of concern to clinicians.

This study retrospectively analyzed the effect of treatment with propranolol for IH and examined the optimal timing for intervention. The optimal timing of treatment with oral propranolol for IH was determined for the first time using receiver operating characteristic (ROC) curve analysis. The aim of this study was to offer recommendations for the early treatment with oral propranolol for IH.

## Methods

### Study design and population

This retrospective study included patients with IH treated with propranolol at the West China Hospital of Sichuan University between January 2014 and December 2021. This study was approved by the Ethics Committee of West China Hospital of Sichuan University (number: 2017414) and was carried out in accordance with the Helsinki Declaration. Informed consent was waived due to the retrospective design of the study and it involved no more than minimal risk to the children. Without a waiver of informed consent, the study could not have been conducted. The inclusion criteria were as follows: (1) age at 1 to 6 months (corrected age for premature infants) with proliferative IH requiring systemic oral propranolol; (2) receiving treatment with oral propranolol for at least 6 months; (3) superficial or mixed IH; (4) IH causing functional impairment (including vision, diet, and hearing), located in cosmetically sensitive areas, or accompanied by ulceration and/or bleeding; and (5) the minimum diameter of the lesion met the criteria of 1.5 cm for facial lesions and 3 cm for nonfacial lesions (if ulceration was present, the minimum diameter was 1.5 cm). The exclusion criteria were as follows: (1) patients with contraindications to oral propranolol, such as allergies to propranolol, severe bradycardia, and bronchial asthma; (2) patients who have received any previous treatment, including corticosteroids, propranolol, topical timolol, and other treatment; (3) patients with incomplete data and lost to follow-up during the 6 to 12 months of continuous oral propranolol; (4) patients who discontinued treatment within the first 6 months or stopped medication for over 1 month due to non-compliance; and (5) patients unable to oral propranolol for other reasons. For patients with multiple IH lesions, the most significant IH (typically the largest or the most severe lesion) was chosen for detailed analysis.

Clinical data were collected for patients, including sex, age, height, weight, IH classification, IH morphology, IH location, rebound, ulceration, time to treatment success, and adverse events. Digital photographs were taken for patients at the start of treatment and during follow-up to record the size and color of the IH, complemented by ultrasound examinations throughout the treatment course. Treatment outcome was evaluated by two specially trained researchers (KZ and QT) using ultrasound and/or digital photographs. Reliability was assessed using the intraclass correlation coefficient, and any disagreements were resolved through consultation. High-risk IHs were differentiated according to the guideline for the diagnosis and treatment of hemangiomas and vascular malformations (2024 edition) in china [12].

All patients received treatment for a minimum of 6 months and followed up for at least 3 months after propranolol discontinuation. Oral propranolol was initiated at a dosage of 1.0 mg/kg/day, administered three times daily for 1 week, and then increased to 2.0 mg/kg/day three times daily from the second week. The dose of propranolol was adjusted in accordance with body weight (weekly for 2 weeks, then every other week for 2 weeks, and then monthly thereafter). The treatment with propranolol was tapered and discontinued on the complete or nearly complete resolution of IH, or if IH no further improvement (for 3 months of observation) was observed after treatment at month 6.

The primary outcome measure was treatment success with oral propranolol at months 6 to 12. The secondary outcome measure was the treatment success time with oral propranolol, the incidence of ulceration, and the rebound rate. Treatment success was defined as complete resolution or nearly complete resolution of IH. Nearly complete resolution was defined as minimal telangiectasia, erythema, skin thickening, and/or soft tissue swelling [13]. Patients who discontinued treatment or used alternative treatment within the 6 to 12 months were classified as treatment unsuccess. After propranolol discontinuation, an IH appearance regrowth of more than 20% (including changes in color and/or volume) was classified as rebound. All adverse events were recorded in accordance with version 4.0 of the Standard for Common Terminology for Adverse Events, which recorded adverse events during the first 6 months of treatment. The causal relationship of adverse events was determined by research team.

This study included 207 patients with IH, including 157 females and 50 males. One hundred and sixteen patients with IH were treatment success with oral propranolol at months 6 to 12. Patients were further categorized into three subgroups: segmental IH (*n* = 85), high-risk IH (*n* = 130), and nonsegmental and non-high-risk IH (*n* = 39). Treatment success was observed in 44 cases of segmental IH, 67 cases of high-risk IH, and 24 cases of nonsegmental and non-high-risk IH.Three patients with high-risk IH were lost to follow-up after treatment at month 12. Thirteen patients were discontinued treatment or used alternative therapies within the 6 to 12 months (3 cases of segmental IH, 11 cases of high-risk IH, and 2 cases of nonsegmental and non-high-risk IH).

### Statistical analysis

Data were summarized using frequencies (percentages) and medians (interquartile ranges, IQRs). Qualitative variables were compared between groups via the χ^2^ test or Fisher’s exact test, while quantitative variables were analyzed via the Mann‒Whitney U test. Multivariate analysis was conducted on the variables. The odds ratio (OR) and 95% confidence interval (95% CI) were calculated. Bonferroni correction was used for multiple comparisons. The optimal cutoff value for the treatment success with oral propranolol was determined through ROC curve analysis. The Youden index was used to assess the optimal cutoff value. Statistical analysis was performed via SPSS 22.0 software.

## Results

### Patients data

The median age at treatment initiation was 61.0 days (IQR, 45.0–90.0 days).Twenty-six patients (12.6%) with IH exhibited ulceration, 20 patients (10.5%) experienced IH rebound, and 93 patients (44.9%) occurred adverse events, including one severe adverse event. Additional information is provided in Table 1. Multivariate analysis showed that age at treatment initiation (*P* < 0.001), high-risk IH (*P =* 0.002), and segmental IH (*P =* 0.012) were independent risk factors for treatment unsuccess with oral propranolol at months 6 to 12 (Table 2).

**Table 1:** Demographics, clinical characteristics and treatment of patients with IH

Characteristics	All patients with IH (n = 207)
Sex	
Male	50 (24.2%)
Female	157 (75.8%)
Age, median (IQR), day	61.0 (45.0–90.0)
Weight, median (IQR), kg	5.9 (5.0-7.2)
Height, median (IQR), cm	58.0 (55.0–61.0)
Born prematurely	52 (25.1%)
Location	
Head and face	103 (49.8%)
Neck, trunk, and extremity	104 (50.2%)
Morphologic subtype	
Localized	103 (49.8%)
Indeterminate	19 (9.2%)
Segmental	85 (41.0%)
Description	
Superficial	63 (30.4%)
Mixed	144 (69.6%)
High-risk IH	130 (60.8%)
Non-high-risk and nonsegmental IH	39 (18.8%)
Ulceration	26 (12.6%)
Rebound	20/191 (10.5%)
PHACE syndrome	7 (3.4%)
Adverse events	93 (44.9%)
Loss of follow-up after treatment at month 12	3 (1.4%)

*IH* Infantile hemangioma, *IQR* Interquartile range

**Table 2:** Multivariate analysis of factors associated with treatment success and treatment unsuccess groups for IH

Characteristics	Treatment success group (n = 116)	Treatment unsuccess group (n = 91)	Multivariate analysis	
			OR (95% CI)	*P* value
Female	93 (80.2%)	64 (70.3%)	0.570 (0.271–1.196)	0.137
Born prematurely	36 (30.0%)	16 (17.6%)	0.954 (0.432–2.106)	0.907
Age, median (IQR), day	55.0 (38.0–67.0)	80.0 (55.0-101.0)	1.033 (1.021–1.046)	< 0.001*
Location				
Head and face	60 (51.7%)	43 (47.3%)	Reference	Reference
Neck, trunk, and extremity	56 (48.3%)	48 (52.7%)	1.727 (0.793–3.763)	0.169
Description				
Superficial	36 (31.0%)	27 (29.7%)	Reference	Reference
Mixed	80 (69.0%)	64 (70.3%)	1.019 (0.474–2.190)	0.961
High-risk IH	67 (55.8%)	63 (69.2%)	3.429 (1.550–7.587)	0.002*
Segmental IH	44 (37.9%)	41 (45.1%)	2.158 (1.226–5.173)	0.012*

*CI* Confidence interval, *IH* Infantile hemangioma, *IQR* Interquartile range, *OR* Odds ratio

*P < 0.05

### ROC curve analysis

**Fig. 1:**
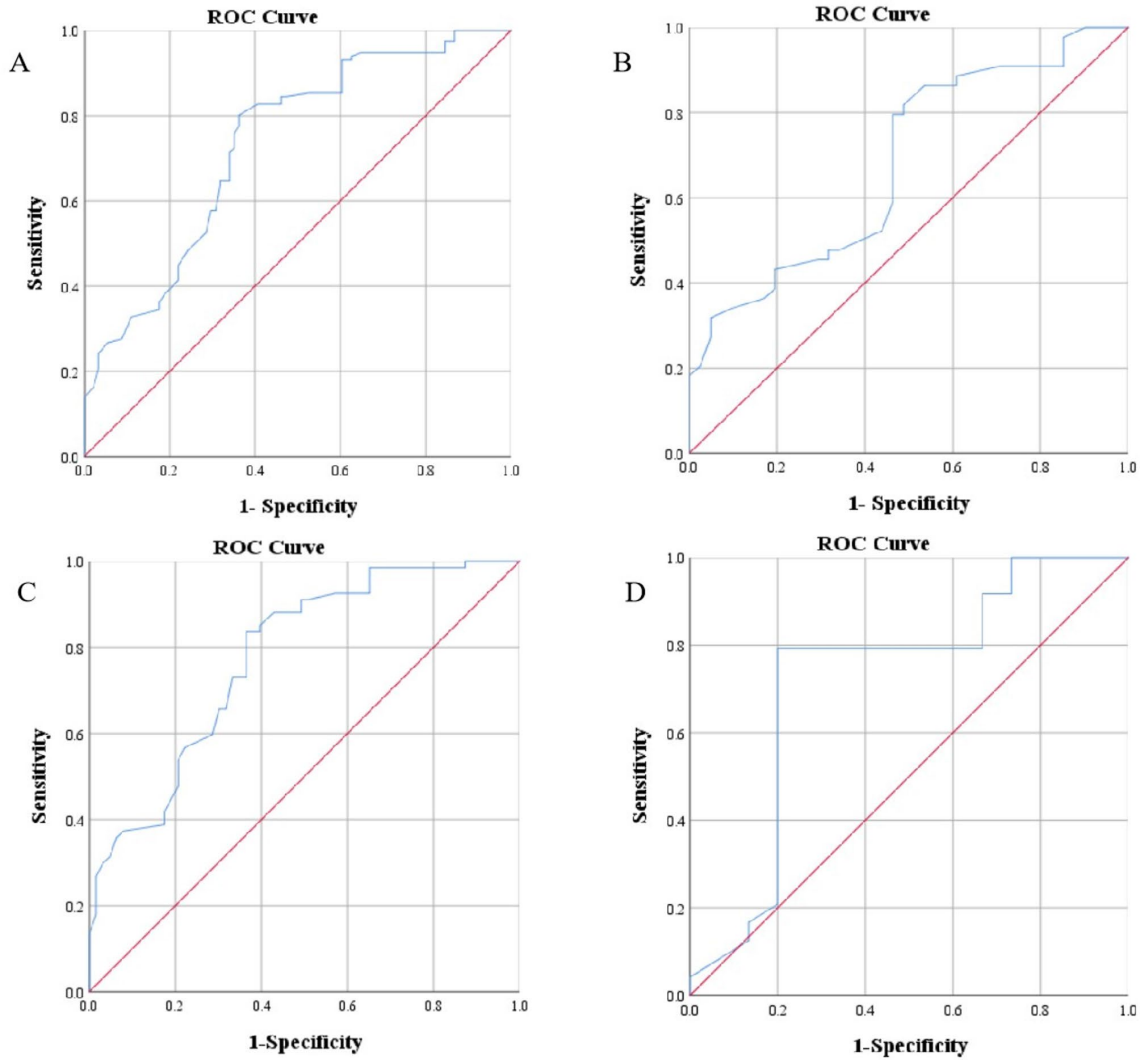
ROC curve analysis of different subgroups. A: The AUC for all patients with IH was 0.733 (95% CI, 0.664–0.802), and the cutoff value was 69.5 days. B: The AUC of patients with segmental IH was 0.680 (95% CI, 0.566–0.794), and the cutoff value was 65.5 days. C: The AUC of patients with high-risk IH was 0.776 (95% CI, 0.697–0.855), and the cutoff value was 65.5 days. D: The AUC of patients with non-high-risk and nonsegmental IH was 0.721 (95% CI, 0.537–0.904), and the cutoff value was 93.5 days

For all patients with IH, the area under the curve (AUC) was 0.733 (95% CI, 0.664–0.802), and the cutoff value was 69.5 days (sensitivity = 80.2%, specificity = 63.7%). For patients with segmental IH, the AUC was 0.680 (95% CI, 0.566–0.794), and the cutoff value was 65.5 days (sensitivity = 79.5%, specificity = 53.7%). For patients with high-risk IH, the AUC was 0.776 (95% CI, 0.697–0.855), and the cutoff value was 65.5 days (sensitivity = 83.6%, specificity = 63.5%). For patients with nonsegmental IH and non-high-risk IH, the AUC was 0.721 (95% CI, 0.537–0.904), and the cutoff value was 93.5 days (sensitivity = 79.2%, specificity = 80.0%) (Fig. 1).

### Subgroup analysis

Based on the ROC curve results, all patients were divided into the age < 69.5 days group (*n* = 126) and the age > 69.5 days group (*n* = 81). Three and ten patients were discontinued treatment or used alternative therapies within the 6 to 12 months in the age < 69.5 days group and the age > 69.5 days group, respectively. In the age < 69.5 days group and the age > 69.5 days group, treatment success was observed in 93 patients (73.8%) and 23 patients (28.4%), respectively (OR, 7.107; 95% CI, 3.803–13.281; *P* < 0.001). The median treatment success time in the age < 69.5 days group was 11.5 months (IQR, 9.1–12.0 months), which was significantly shorter than the 15 months (IQR, 11.8–20.5 months) in the age > 69.5 days group (*P* < 0.001). Additional information is detailed in Table 3.

**Table 3:** Primary and secondary outcomes between the age < 69.5 days group and the age > 69.5 days group for all patients with IH

Outcomes	Age < 69.5 days group (*n* = 126)	Age > 69.5 days group (*n* = 81)	*P* value	OR (95% CI)
Treatment success at month 6 to 12	93 (73.8%)	23 (28.4%)	< 0.001*	7.107 (3.803–13.281)
Time to treatment success (months)	11.5 (9.1–12.0)	15.0 (11.8–20.5)	< 0.001*	-
Ulceration	15 (11.9%)	11 (13.6%)	0.723	0.860 (0.374–1.979)
Rebound	15/122 (12.3%)	5/69 (7.2%)	0.274	1.794 (0.623–5.171)

*CI* Confidence interval, *IH* Infantile hemangioma, *OR* Odds ratio

*P < 0.0125

Patients with segmental IH were divided into two groups: the age < 65.5 days (*n* = 54) and the age > 65.5 days (*n* = 31). Three patients were discontinued treatment or used alternative therapies within the 6 to 12 months in the age > 65.5 days group. In the age < 65.5 days group and the age > 65.5 days group, treatment success was observed in 35 patients (64.8%) and 9 patients (29.0%), respectively (OR, 4.503; 95% CI, 1.732–11.710; *P* = 0.001). The median treatment success time in the age < 65.5 days group was 11.6 months (IQR, 7.3–18.7 months), which was shorter than the 17.5 months (IQR, 11.4–19.2 months) in the age > 65.5 days group (*P* = 0.039). Additional information is detailed in Table 4.

**Table 4:** Primary and secondary outcomes between the age < 65.5 days group and the age > 65.5 days group for segmental IH

Outcomes	Age < 65.5 days group(n = 54)	Age > 65.5 days group (n = 31)	*P* value	OR (95% CI)
Treatment success at month 6 to 12	35 (64.8%)	9 (29.0%)	0.001*	4.503 (1.732–11.710)
Time to treatment success (months)	11.6 (7.3–18.7)	17.5 (11.4–19.2)	0.039	-
Ulceration	8 (14.8%)	5 (16.1%)	0.871	0.904 (0.268–3.052)
Rebound	5 (9.3%)	0	0.160	-

*CI* Confidence interval, *IH* Infantile hemangioma, *OR* Odds ratio

*P < 0.0125

Patients with high-risk IH were divided into two groups: the age < 65.5 days group (*n* = 79) and the age > 65.5 days group (*n* = 51). One and two patients were lost to follow-up after treatment at month 12 in the age < 65.5 days group and the age > 65.5 days group, respectively. Two and nine patients were discontinued treatment or used alternative therapies within the 6 to 12 months in the age < 65.5 days group and the age > 65.5 days group, respectively. In the age < 65.5 days group and the age > 65.5 days group, treatment success was observed in 56 patients (70.9%) and 11 patients (21.6%), respectively (OR, 8.854; 95% CI, 3.879–20.206; *P* < 0.001). The median treatment success time in the age < 65.5 days group was 11.5 months (IQR, 8.3–14.5 months), which was significantly shorter than the 16.5 months (IQR, 12.0–18.7 months) in the age > 65.5 days group (*P* < 0.001). Additional information is detailed in Table 5.

**Table 5:** Primary and secondary outcomes between the age < 65.5 days group and the age > 65.5 days group for high-risk IH

Outcomes	Age < 65.5 days group (*n* = 79)	Age > 65.5 days group (*n* = 51)	*P* value	OR (95% CI)
Treatment success at month 6 to 12	56 (70.9%)	11 (21.6%)	< 0.001*	8.854 (3.879–20.206)
Time to treatment success (months)	11.5 (8.3–14.5)	16.5 (12.0-18.7)	< 0.001*	-
Ulceration	14 (17.7%)	6 (11.8%)	0.358	1.615 (0.577–4.521)
Rebound	10/76 (13.2%)	3/40 (7.5%)	0.543	1.869 (0.484–7.219)

*CI* Confidence interval, *IH* Infantile hemangioma, *OR* Odds ratio

*P < 0.0125

Patients with non-high-risk and nonsegmental IH were divided into the age < 93.5 days group (*n* = 22) and the age > 93.5 days group (*n* = 17). Two patients were discontinued treatment or used alternative therapies within the 6 to 12 months in the age > 93.5 days group. In the age < 93.5 days group and the age > 93.5 days group, treatment success was observed in 19 patients (86.4%) and 5 patients (29.4%), respectively (OR, 15.200; 95% CI, 3.058–75.574; *P* = 0.001). The median treatment success time in the age < 93.5 days group was 11.5 months (IQR, 10.6–12.0 months), which was shorter than the 13.5 months (IQR, 8.4–18.0 months) in the age > 93.5 days group (*P* = 0.079). Additional information is detailed in Table 6.

**Table 6:** Primary and secondary outcomes between the age < 93.5 days group and the age > 93.5 days group for non-high-risk and nonsegmental IH

Outcomes	Age < 93.5 days group (*n* = 22)	Age > 93.5days group (*n* = 17)	*P* value	OR (95% CI)
Treatment success at month 6 to 12	19 (86.4%)	5 (29.4%)	0.001*	15.200 (3.058–75.574)
Time to treatment success (months)	11.5 (10.6–12.0)	13.5 (8.4–18.0)	0.079	-
Ulceration	2 (9.1%)	3 (17.6%)	0.636	0.467 (0.069–3.168)
Rebound	4 (18.2%)	0	0.118	-

*CI* Confidence interval, *IH* Infantile hemangioma, *OR* Odds ratio

*P < 0.0125

## Discussion

This study offers insights into the optimal timing of treatment with oral propranolol for IH. Our study introduces innovation in statistical methods by being the first to utilize the ROC curve to determine the specific cutoff value of oral propranolol for IH, differing from the approach employed by Léauté-Labrèze et al. [14]. A subgroup analysis was conducted to determine the optimal age at treatment initiation with propranolol for segmental and high-risk IH. The results suggest that early treatment with propranolol is crucial for these patients. This study enhances the understanding of both specialist and nonspecialist physicians regarding the appropriate timing of treatment with propranolol for IH, thereby facilitating better anticipatory guidance.

**Fig. 2:**
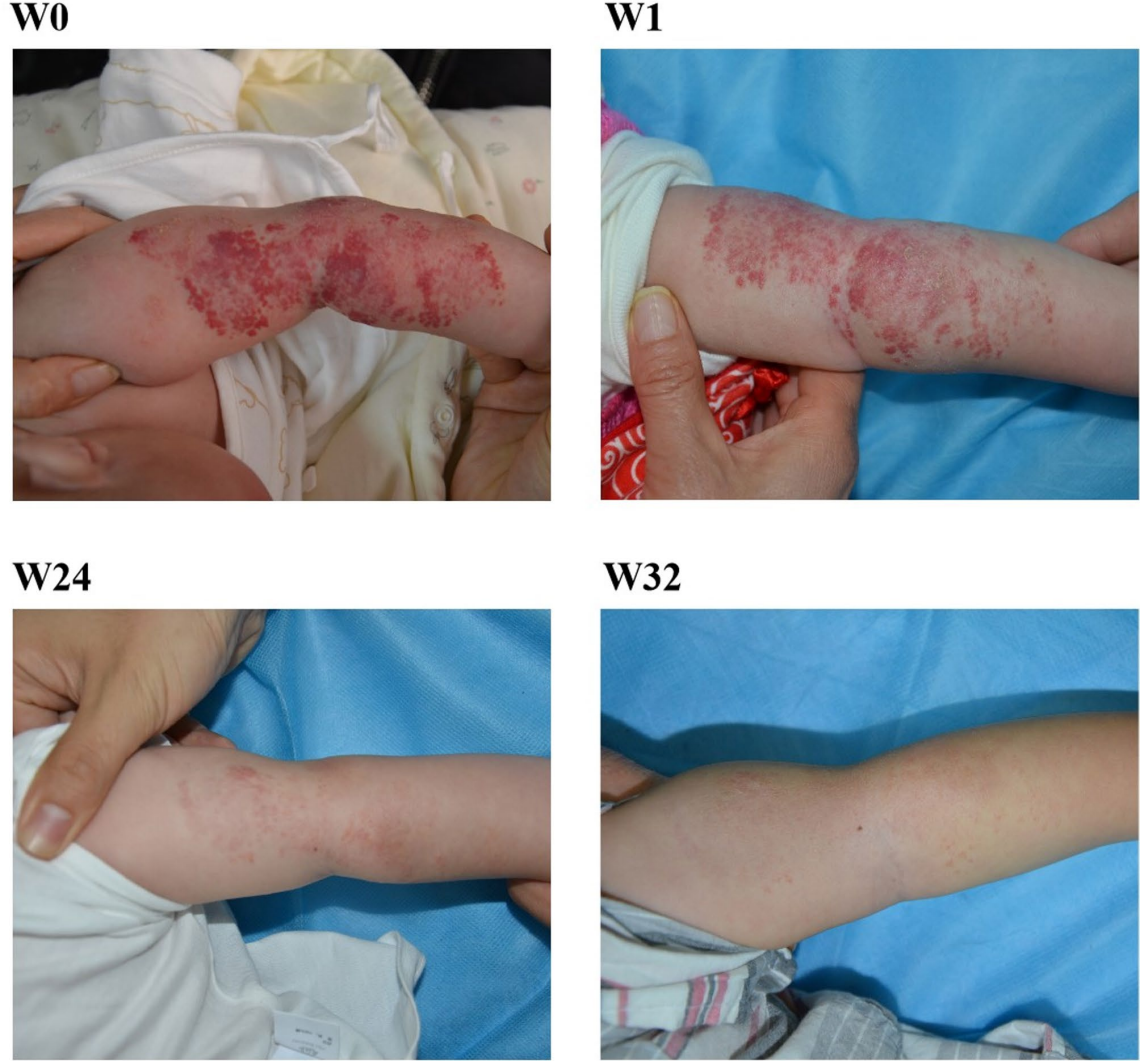
A 62-day-old girl with segmental IH in the left upper limb. A nearly complete resolution of the IH was noted at month 6

The optimal threshold of 69.5 days was derived from the ROC curve of the all patients with IH. Subgroup analysis indicated that thresholds of 65.5 days and 93.5 days were suitable for specific populations. Therefore, a universally applicable threshold of 69.5 days was selected as the key threshold. In clinical practice, the identified age thresholds can guide timely intervention. For infants with IH, initiating oral propranolol before 69.5 days of age significantly improves treatment success (Fig. 2). The treatment success rate was 7.1 times higher before 69.5 days of age compared to after. Patients with high-risk IH or segmental IH requires more earlier intervention, as treatment delays drastically reduce the likelihood of success. In contrast, non-high-risk and nonsegmental IH allows for more flexibility, with treatment remaining effective up to 93.5 days of age, but prompt treatment may be better. Pediatricians should perform early screening, rapidly stratify risk, and urgently refer high-risk cases. When counseling parents, it is important to emphasize that earlier treatment initiation shortens the duration of therapy and increases the probability of success, thereby supporting informed decision-making. This tailored approach optimizes clinical outcomes while remaining adaptable to real-world clinical settings.

This study partially differs from the other studies. The primary outcome measure of their study was treatment success after 6 months of continuous propranolol [13, 14], whereas our study focused on treatment success during 6 to 12 months. Because most IH resolution after oral propranolol within 6 to 12 months. Previous reports were based on the population data of oral propranolol at 1 mg/kg/day or 3 mg/kg/day [13–15], but no population data of 2 mg/kg/day. The aim of our study was to evaluate oral propranolol at 2 mg/kg/day to address this gap. The potential effects of different propranolol doses on the timing of IH treatment were investigated.

**Fig. 3:**
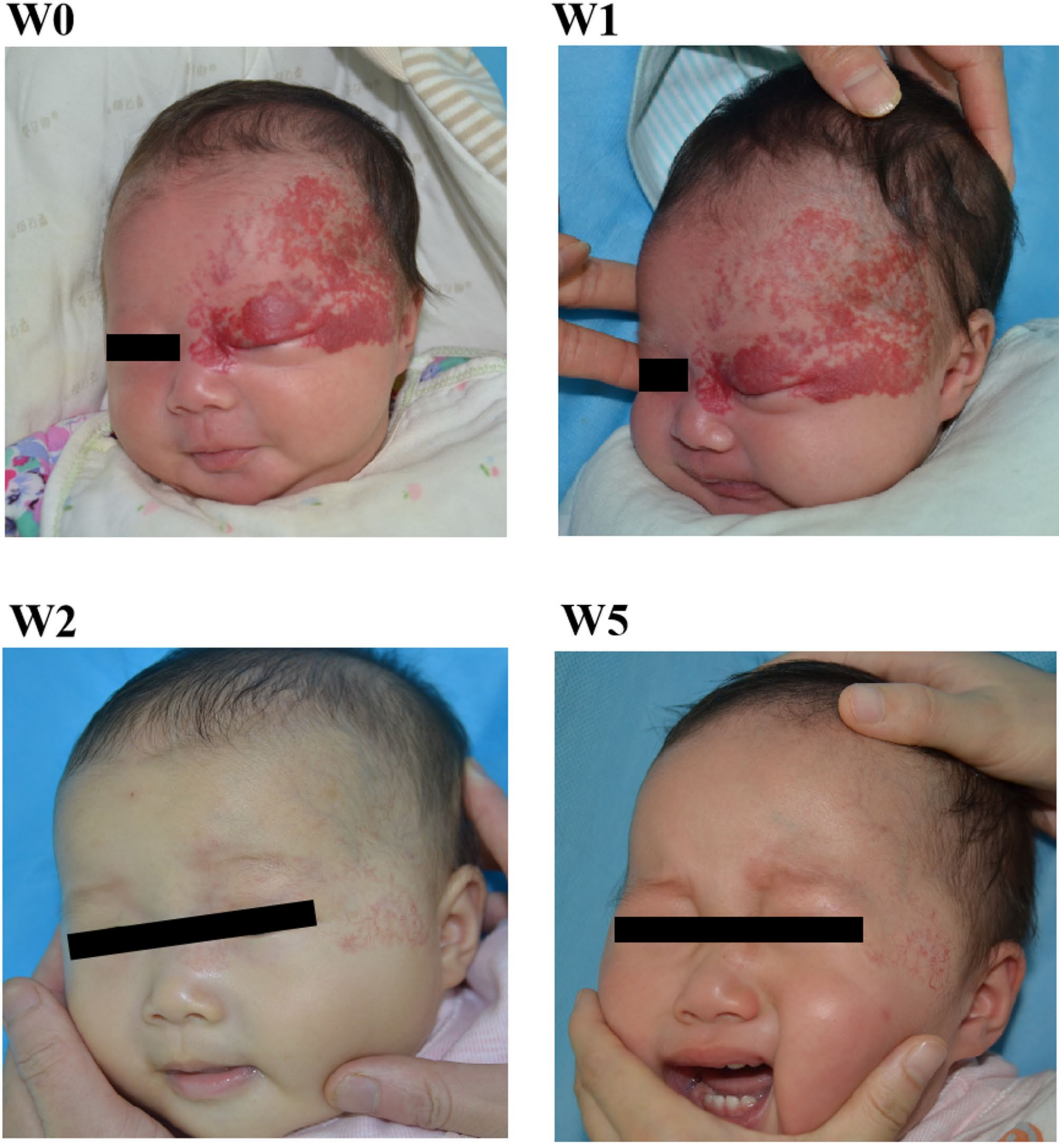
A 39-day-old girl with facial segmental IH. A nearly complete resolution of the IH was noted at month 6

Baselga et al. [16]reported that the success rate of high-risk IH can reach 76% at 12 months of age with oral propranolol, whereas the success rate with oral propranolol in our study was only 51.5%, and the treatment success rate before 65.5 days of age at treatment initiation reached 70.9%. The failure rate before 65.5 days of age at treatment initiation is roughly one-tenth that of those treated after 65.5 days, clearly demonstrating that early treatment significantly enhances the success rate. (Fig. 3). The timing assessment of oral propranolol for IH treatment focuses not only on efficacy but also on safety and tolerability in patients [2]. In this study, there were no fatalities among all IH patients, and only one severe adverse event occurred (not meeting the discontinuation criteria). Mild to moderate adverse events were more common. IH rebound is prone to occur in patients with a younger age at treatment initiation, which might be attributed to poor family compliance and premature drug withdrawal.

Many parents were concerned about the lesion within one month after the birth of their child. However, some primary care physicians informed parents that IH was benign tumor and resolution spontaneously, opting for an observational approach. This has led to delayed treatment for certain patients, affecting their prognosis [14]. Studies indicated that patients with IH usually receive specialist treatment around 5 months of age, a point at which most IHs have already progressed significantly, often resulting in less optimal treatment outcomes [17, 18]. Delaying treatment increases the risk of complications like ulceration and raises the likelihood of irreversible skin damage [14]. Therefore, the timing of treatment with propranolol is particularly crucial. The optimal timing of oral propranolol for IH remains a topic of interest, warranting future prospective, multicenter, and large-sample studies.

Our study included patients with IH who received alternative treatment within the 6 to 12 months. Excluding patients who required alternative interventions may introduce bias in estimating the true efficacy of oral propranolol for IH. These patients often chose alternative treatment options due to inadequate responses to propranolol (such as stable lesion size or the development of drug resistance) and they carry a higher risk of treatment failure. Therefore, excluding these patients would result in the study predominantly including cases sensitive to propranolol, potentially overestimating the actual success rates of treatment with propranolol.

### Limitations

This study has several limitations. First, this was a retrospective study and pre-selection bias due to the nonresponse to the survey. Second, a limitation that weakens the generalizability of the current findings is the single-center of the study.Finally, the subjects of the study were infants, and the adverse reactions after oral propranolol were mostly subjective descriptions provided by parents, with significant variations in accuracy.

## Conclusion

The initiation of treatment for IH with oral propranolol before 70 days of age significantly improves success rate and shortens treatment duration. Early intervention is crucial for high-risk IH and segmental IH. These findings underscore the importance of individualized treatment timing based on subtype and risk level, optimizing clinical diagnosis and treatment processes and improving overall outcomes.

## Data Availability

The data pertinent to this study are accessible through the corresponding author.

## Abbreviations

AUC: Area under the curve
CI: Confidence interval
IH: Infantile hemangioma
IQR: Interquartile range
OR: Odds ratio
ROC: Receiver operating characteristic
